# S100B levels are affected by older age but not by alcohol intoxication following mild traumatic brain injury

**DOI:** 10.1186/1757-7241-21-52

**Published:** 2013-07-06

**Authors:** Olga Calcagnile, Anders Holmén, Michelle Chew, Johan Undén

**Affiliations:** 1 Department of Pediatric Medicine, Hallands Hospital, Halmstad 30185, Sweden; 2 Department of Research and Development, Hallands Hospital, Halmstad 30185, Sweden; 3 Department of Anaesthesia and Intensive Care and Institute of Clinical Sciences Malmö, Lund University, Hallands Hospital, Halmstad 30185, Sweden; 4 Department of Intensive Care and Perioperative Medicine and Institute of Clinical Sciences Malmö, Lund University, Skanes University Hospital, Malmo 20502, Sweden

## Abstract

**Introduction:**

Biomarkers of brain damage and head injury are potentially useful tools in the management of afflicted patients. Particularly S100B has received much attention and has been adapted into clinical guidelines. Alcohol intoxication and higher age (65 years and over) have been used as risk factors for serious complications following head injury. The effect of these factors on S100B levels has not been fully established in a relevant patient cohort.

**Methods:**

We prospectively included 621 adult patients with mild traumatic brain injury (TBI) and S100B sampling. Mild TBI was defined as Glasgow Come Scale 14–15 with loss of consciousness and/or amnesia, but without high-risk factors for intracranial complications. These patients would normally require CT scanning according to local and most international guidelines. S100B was sampled within 3 hours following trauma.

**Results:**

280 patients (45%) were intoxicated by alcohol. Alcohol intoxication had no effect on S100B levels (p = 0.65) and the performance of S100B remained unchanged in these patients. 115 patients (22%) were 65 years or older with elevated S100B levels being more common in this group compared to patients under 65 (p = 0.029). Although the sensitivity of S100B was unchanged in older patients, the specificity was poorer.

**Conclusion:**

S100B can be used reliably in mild TBI patients with alcohol intoxication. The clinically utility of S100B in older patients may be limited by very poor specificity leading to only a small decrease in CT scanning.

## Introduction

Biochemical markers are used as screening and diagnostic tools in many clinical scenarios. Recently, biomarkers for diagnosis and prognosis of brain injury have developed [1]. These may allow faster and more accurate management of brain disease, similarly to biomarkers used in other organ systems. Traumatic brain injury (TBI) is a leading cause of death, especially in younger individuals [2]. Management usually involves computed tomography of the brain to detect lesions that may need neurosurgical or medical intervention. However, most of there injuries are not serious in nature, often called mild TBI or minor head injury. Serious complications following mild TBI are rare, with approximately 5% displaying traumatic CT pathology and less than 1% needing specific intervention [3,4]. Despite this, CT is recommended in these patients due to the seriousness of the complications [5-7]. Attempts to reduce CT use have been based upon aspects of patient history and clinical examination. However, these may be inaccurate in any patients, especially in those with head trauma or brain injury. An objective biomarker would therefore we welcomed in the management of these patients.

S100B is a small calcium-binding protein weighing approximately 21 kDa, predominantly expressed by glia cells. When brain tissues and/or the blood brain barrier (BBB) are damaged, S100B is released and can be detected in the peripheral blood. Many studies have shown the potential of S100B as a biomarker in brain injuries [8-10]. In particular, the potential of S100B to reduce unnecessary CT scans following mild TBI has received considerable attention. Recently, international guidelines have been published including S100B as a management option [11].

Many patients with mild TBI are intoxicated by ethanol [12]. Although some studies have shown little effect of alcohol on S100B levels [12], others have shown conflicting results [13]. This aspect is important if the biomarker is to function effectively in this population. False high S100B due to alcohol intoxication would limit the CT-reducing ability of the biomarker.

Children have higher levels of S100B than adults [14]. However, levels of S100B in elderly patients following mild TBI have not been investigated. Since older age (most often defined as over 65 years of age) is often included as a risk factor for complications after TBI [15,16], this aspect is also of importance.

The aim of this study is to investigate the relationship of older age and alcohol intoxication to serum S100B levels following mild TBI in a large prospective cohort.

## Methods

### Study setting and cohort population

We undertook a prospective study in Halmstad Regional hospital, Sweden, from June 2008 to December 2012. Our hospital is a level II trauma centre with 24-hour emergency care, anesthesiology, radiology, surgery and intensive care. Approximately 6 months prior to the study, local guidelines for management of mild TBI, including S100B sampling, were introduced into clinical practice.

We consecutively enrolled all adult patients with mild TBI and subsequent S100B sampling. Inclusion criteria were; adult patients with trauma to the head with GCS 14–15 during examination and loss of consciousness < 5 minutes or amnesia. Exclusion criteria were; age less than 18 years, focal neurological deficit, therapeutic anticoagulation or haemophilia, radiographically demonstrated skull fracture, clinical signs of depressed skull fracture or skull base fracture, posttraumatic seizure, shunt-treated hydrocephalus, multiple organ trauma and patients where serum sampling for S100B was taken more than 3 hours post-injury.

Patient age at time of trauma and alcohol intoxication (yes/no based upon patient history and examination) was prospectively documented. Determination of blood alcohol levels was determined based upon the discretion of the treating physician. The age limit of 65 years or older was the pre-determined cut-off for analysis based upon published management rules [11,16].

The study was approved by the regional ethical board (approval number 19/2007).

### Blood sampling and biochemical analysis

A 5 ml blood sample was drawn from patient’s cubital vein in the ED. Samples were analyzed with the fully automated Elecsys® S100 (Roche AB) at the Clinical Chemistry Department of Halmstad Regional hospital, Sweden. Roche AB report a range between 0.005 μg/L and 39 μg/L and a within-series coefficient of variance of <2.1%. Based on the available evidence at this time, we chose a cut-off level for normal levels of less than 0.10 μg/L and a window of sampling of 3 hours from the time of the accident [17,18]. Lab results were available to treating physicians within 1 hour after sampling.

### CT examinations

Cranial CT scans were performed with a GE VCT Ligthspeed 64 multislice detector with a 0,625/0,625 mm, 0,5 seconds rotation time and pitch of 0,531:1. 10 mm thick slices were used as part of the standard CT protocol for these patients. CT scans are always analysed by a board certified radiologist and confirmed by a consultant radiologist. Since S100B was used clinically, radiologists were not blinded to S100B results. A CT scan was considered positive if any sings of cranial (skull fracture) or intracranial pathology (hematoma, air or contusion) were present.

### Follow-up

Patients were followed up after 3 months post-trauma by questionnaire. This contained information regarding clinical symptoms suggestive of intracranial complications and included additional (new) CT scans and/or exposure to the health care system. Patients who were lost to follow-up were checked by examination of medical records and national mortality databases for signs of intracranial complications or death.

### Statistic analysis

Data was registered on an Excel® file. The difference in S100B levels between age groups and intoxicated/sober patients were calculated with a Mann–Whitney test. A non-parametric test was chosen due to a skewed distribution of data.

## Results

Between June 2008 and December 2012, we enrolled 621 patients with mild TBI and S100B levels. 351 patients had CT scans as part of their management (322 with S100B levels equal to or higher than 0.10 μg/L and 29 patients with S100 levels lower than 0.10 μg/L). 513 (83%) of patients had successful and complete follow-up including 242 (90%) of the 270 patients not receiving an initial CT. No patients showed any new signs of intracranial complications. A total of 29 patients had cranial CT pathology but only 26 (4.7%) of these showed traumatic abnormalities (isolated skull fracture n = 3, cerebral contusions n = 9, acute subdural hematoma n = 3, intracranial air n = 1, combinations of traumatic intracranial findings n = 10). The remaining 3 patients had non-traumatic findings unrelated to the injury.

280 patients (45%) were intoxicated by alcohol and a blood alcohol level was determined in 197 patients. All patients who had blood drawn for alcohol analysis were clinically suspected of intoxication and all had measureable levels. 115 patients (19%) of the total cohort were 65 years or older.

**Figure 1:**
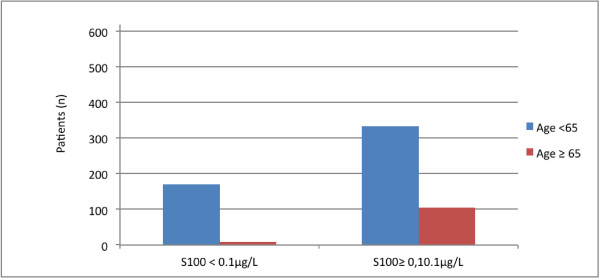
Bar graph showing the relationship between age and S100B levels.

**Figure 2:**
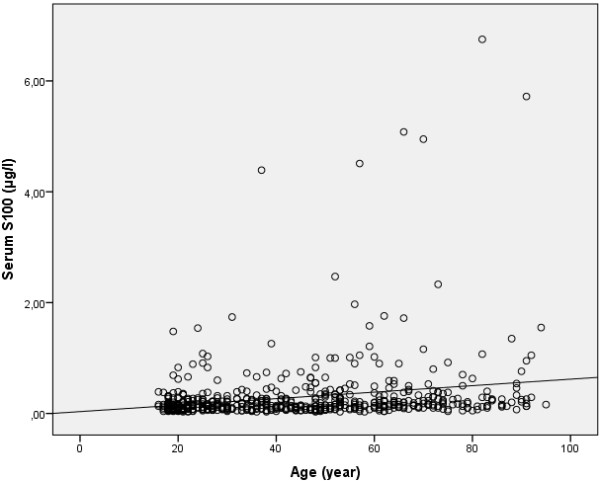
**Scatter plot showing S100B levels verses age in patients following mild TBI.** Spearman’s rho 0,295, p = 0,000.

180 patients (29%) had a S100B level lower than 0.10 μg/L: 171 of these (95%) were younger than 65 years and only 9 patients (5%) were older or equal than 65 years of age. Figure 1 shows a bar graph of age and S100B levels in the study population. 441 patients (71%) showed a S100B level higher or equal to 0.10 μg/L: 335 (76%) of these were younger than 65 years old and 106 patients (24%) were older or equal than 65 years old. The difference in S100B levels between the age groups was significant (p = 0.029), see Table 1. A scatter plot of age verses S100B levels is shown in Figure 2.

**Table 1:** S100B levels in 621 patients with mild TBI

	**S100b < 0.10 μg/L**	**Median S100b**	**S100b ≥ 0.10 μg/L**	**Median S100b**
		**μg/L**		**μg/L**
**<65 years**	171 patients	0.07	335 patients	0.19
		(0.03-0.09)		(0.10-4.51)
**≥65 years**	9 patients	0.07	106 patients	0.23
		(0.04-0.09)		(0.10-6.75)
**No Alcohol intoxication**	106 patients	0.07	235 patients	0.20
		(0.03-0.09)		(0.10-6.75)
**Alcohol intoxication**	74 patients	0.07	206 patients	0.20
		(0.03-0.09)		(0.10-4.51)

Table shows the number of patients (and interquartile range in brackets) with S100B levels above and below 0.10 μg/L and the number of patients above or below 65 years of age and with or without alcohol intoxication. The difference in S100B levels between patients 65 and over with patients under 65 years was significant (p = 0.029) and the difference between intoxication and no intoxication was not significant (p = 0.65).

**Figure 3:**
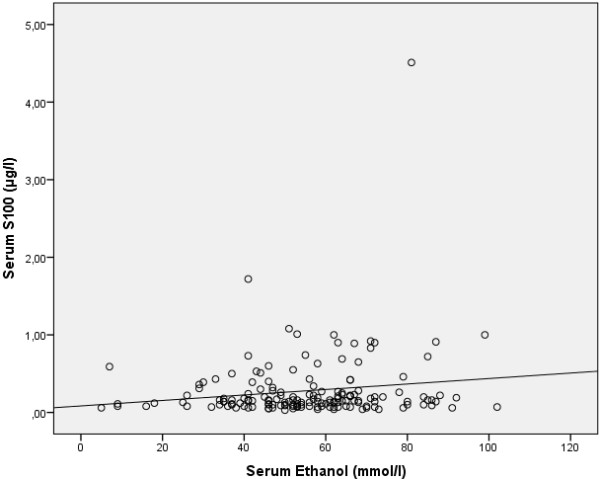
**Scatter plot showing S100B levels verses serum ethanol levels in patients following mild TBI.** Spearman’s rho 0,092, p = 0,249.

206 of the 280 patients (74%) who were intoxicated by alcohol had a S100B level higher or equal to 0.10 μg/L. 235 of the 338 (70%) patients without alcohol intoxication had elevated S100B levels. There was no statistical difference in S100B levels between those patients with and without intoxication (p = 0.65), see Table 1. For the 197 patients where serum ethanol levels were determined, a scatterplot was created, see Figure 3. 10 patients were both 65 years or older and intoxicated by alcohol.

S100B had a sensitivity of 100% and a specificity of 30% for CT findings in the entire population. The specificity increased to 35% if only patients younger than 65 years were considered. The positive likelihood ratio that a S100B level higher or equal to 0.10 μg/L would predict a pathological CT was 1.44 for the entire population while it increased to 1.54 if we considered only patients younger than 65 years of age.

## Discussion

The initial management of mild TBI is still under debate. Several guidelines and decision rules, derived from different cohorts from different countries, have been published and are currently used clinically [11,15,16,19]. The introduction of S100B into clinical practice has been shown to improve the management of mild TBI [20] with a reduction of CT scans following these injuries. This has the potential to reduce costs and potential radiation dosages to patients with head injury.

New, updated international guidelines, including blood sampling for S100B, are presently being implemented in Scandinavia [11]. These guidelines recommend S100B sampling in adult patients with loss of consciousness and/or repeated (more than one episode) vomiting if other risk factors are absent. One such risk factor is older age (65 years or older) in combination with anti-thrombocyte medication. As many elderly patients take these medications, the majority of these patients will have this risk factor and not be eligible for S100B sampling. Based upon the results of this study, this approach seems reasonable. Even if the sensitivity of S100B for CT findings was still 100% in elderly patients, the specificity was worse. In practice, this results in a smaller potential reduction in CT scanning after mild TBI and hence a weaker clinical indication for the test. The reason for this observation is unclear. One may speculate that the higher S100B levels observed are merely a reflection of the increased risk of brain injury these patients have following brain trauma. If this is the case, S100B levels may still correctly classify these patients as high risk mild TBI indicating the necessity of a CT scan. Also, older patients often have concurrent chronic disease and may also have neurological disease such as Alzheimer’s disease or Parkinson’s disease. In the present study, non-neurological disease was not registered and the prevalence of neurodegenerative disease was too low to perform any meaningful analysis. It may also be argued that the cut-off for S100B should be higher in older patients. This was, however, not an endpoint of this study. Higher cut-off levels have been shown to be more specific in previous studies [21]. However, the large body of evidence and current clinical practice is focused on the 0.10 μg/L level and is seems reasonable to primarily consider this cut-off although to ensure maximal sensitivity in clinical practice. These issues should be confirmed in future studies.

We found no affect of alcohol on S100B levels, irrespective of whether alcohol intoxication was derived from patient history and clinical examination or from objective blood ethanol levels. This confirms previous observations from another cohort [12] but is somewhat in contrast to other reports [13]. Different methods of S100B analysis may have influenced these conflicting results [22]. The results from this study are based upon a much larger cohort than the previous studies and consider a pragmatic and clinically relevant patient material. This observation is important considering the frequency of alcohol intoxication in these patients. Indeed, in this study, 45% of patients were intoxicated by alcohol.

Considering these results, S100B can be used freely in mild TBI patients with alcohol intoxication. This is naturally welcomed, due to the difficulties of assessing patient history, performing adequate and reliable clinical examination and obtaining a cranial CT scan of intoxicated patients. Although S100B shows a 100% sensitivity for CT findings after mild TBI in all age groups, the performance of the biomarker, specifically the ability of S100B to decrease unnecessary CT scans, will likely be reduced in elderly (65 years or older) patients. Although this is in accordance to recent guidelines [11], this should also be considered in other scenarios. The health economic implications of S100B use in this patient group remains to be shown.

## Conclusion

In patients with mild TBI, S100B is unaffected by alcohol intoxication and may be used effectively in this patient group. Patients aged 65 years and older had higher S100B levels and the overall ability of S100B to reduce CT scans in the elderly may be impaired.

## Competing interests

JU has in previous studies (unconnected with the current study) received S100B analysis kits from Roche AB, Sweden and Diasorin AB, Sweden. JU has previously lectured for Roche AB, Sweden. AH, OC and MC did not have any competing interests.

## Authors’ contributions

JU and OC conceived the study. JU and OC acquired data. JU, AH, MC and JU did data analysis. JU and OC drafted the paper with input from AH and MC. All authors read and approved the final manuscript.
